# Preparation of Self-Curling Melt-Blown Fibers with Crimped Masterbatch (CM) and Its Application for Low-Pressure Air Filtration

**DOI:** 10.3390/polym15163365

**Published:** 2023-08-10

**Authors:** Xiaofang Lin, Minggang Lin, Tan Li, Hao Lu, Huan Qi, Ting Chen, Lili Wu, Chuyang Zhang

**Affiliations:** 1 College of Textile and Clothing Engineering, Soochow University, Suzhou 215123, China; 20214215035@stu.suda.edu.cn (X.L.); tingchen@suda.edu.cn (T.C.); liliwu@suda.edu.cn (L.W.); 2 Institute of Smart & Ecological Textile, Quanzhou Normal University, Quanzhou 362002, China; 107552104255@stu.xju.edu.cn (M.L.); 17369281906@163.com (T.L.); luhao@stu.xju.edu.cn (H.L.); 3 Key Laboratory of Clothing Materials of Universities in Fujian, Quanzhou Normal University, Quanzhou 362002, China; 4 College of Textile and Apparel, Quanzhou Normal University, Quanzhou 362002, China

**Keywords:** melt-blown nonwoven, curling fibers, fluffiness, low pressure filtration, electrostatic decay

## Abstract

Particulate matter (PM) and airborne viruses pose significant threats to both the environment and public health. As the most viable solution to prevent the inhalation of these pollutants, there is an urgent demand for face masks with excellent filtration efficiency and low-pressure drop. In this study, a crimped masterbatch (CM) is added to polypropylene feedstocks to produce curling fibers through melt-blown spinning. These curled fibers exhibit low filtration resistance and effective dust-holding performances when used for air filtration. The effect of adding CM on fiber diameter, pore size, crimp, porosity, roughness, and surface potential was studied. The filtration performance of the materials, including the PM filtration capabilities, recirculation filtration, and loading test performance, were also investigated. The results demonstrate that the degree of fiber crimp can be adjusted by incorporating varying amounts of CM. This curling was caused by the uneven shrinkage that occurred due to variations in thermal contraction between these polymers. The curled fibers created a fluffy structure in the fiber network and modified the distribution of pore sizes within it. Under the same filtration conditions as sodium chloride aerogel, CM–2 (PP:CM 8:2) exhibited similar filtration efficiency (95.54% vs. 94.74%), lower filtration resistance (88.68 Pa vs. 108.88 Pa), higher quality factor (0.035 Pa^−1^ vs. 0.028 Pa^−1^) and better dust holding capacity (10.39 g/m^2^ vs. 9.20 g/m^2^) compared to CM–0 (PP:CM 10:0). After 30 days of indoor storage, the filtration efficiency of CM–2 remained above 94%. The self-curling melt-blown filtration material developed here could potentially be applied in the field of protective masks.

## 1. Introduction

With the rapid development of global industry, airborne pollutants such as particulate matter (PM) and viruses pose huge risks to human health [1]. The WHO (World Health Organization) reports that more than 90% of the world’s population is exposed to contaminated air, which can cause health problems. It is estimated that air pollution will cause more than 500,000 premature deaths each year, and the number of deaths caused by ambient air pollutants is projected to double by 2050 [2,3]. In addition to air pollution, outbreaks of emerging infectious diseases, particularly COVID-19, have resulted in significant casualties and serious impacts on social stability [4]. Typically, viruses are transmitted by direct contact, droplets, and aerosols [5,6]. Wearing masks is the most effective method to prevent inhaling pollutants and spreading viruses. To safeguard the public from pollutants and viruses, there is an urgent need for masks that offer excellent filtration performance and high levels of comfort. Facemasks are typically made of nonwovens. Spunbonded and needle-punched fibers typically have diameters in the range of a few tens of microns, making them suitable only for coarse filtration. These fibers are commonly utilized as the mask’s covering layer or lining. In recent years, there has been extensive research on electrospun filter materials due to their high porosity and low surface density [7,8]. However, there are several disadvantages associated with electrostatically spun materials that limit their further development. These include high-pressure drops, solvent residues, time-consuming production, and difficulties in industrial manufacturing [9,10,11].

Melt-blown nonwovens possess excellent filtration properties due to their high specific surface area, fine fiber diameter, and narrow pore size distribution [12,13,14]. However, the minimum average diameter of melt-blown fibers is 1–2 μm, while electrostatically spun fibers have diameters of 100–500 nm. This significant difference in diameter creates a gap that reduces the probability of capturing PM by melt-blown fibers [15,16]. The filtration efficiency can be improved by increasing the basis weight of melt-blown nonwovens or by incorporating electrostatically spun nanofibers [1,17]. However, this method leads to a substantial increase in the filter resistance. Therefore, it is highly significant to develop filtration materials that can provide both effective protection and comfortable breathing simultaneously. For a long time, chemical fibers have drawn inspiration from natural fibers, particularly wool fibers, with their curly structures [18,19]. The curled fibers create a fluffier web structure when their diameter is consistent. In recent years, twisted fibers have been utilized in nonwoven materials. Hui et al. selected polyurethane (TPU) and polypropylene (PP) to prepare a curly fiber nonwoven using a specially designed rotary-flow mold melt-blown device [20]. Due to the poor compatibility between PP and TPU, the fibers with a curly structure cannot be formed stably during spinning. Li et al. obtained nanofibers with a small diameter (~0.6 μm), which is two orders of magnitude smaller than that of natural wools (~20 μm). The polyvinylidene fluoride (PVDF) nanofibers achieve an ultra-high porosity of 98.7% through self-curling and in situ charging [4]. Zheng et al. prepared a coiled PVDF nanofiber film through electrospinning. The qualification factor increased from 0.0274 Pa^−1^ to 0.0309 Pa^−1^, representing a 12.8% improvement after optimizing the electrospinning parameters. The filtration efficiency and pressure drops were 93.6% and 89.0 Pa, respectively [21]. Although electrospun nanofibers are at the forefront of advanced materials, the time-consuming nature of their production is a serious impediment to their industrialization. The development of crimped melt-blown fibers not only holds great potential for personal protection in the industry but also promotes the application of low-cost, multifunctional melt-blown fibers.

Herein, we propose a one-step strategy to fabricate self-curling melt-blown fibers by adding a crimped masterbatch to polypropylene feedstocks. When combined with the electrostatic electret effect, melt-blown filters exhibit high porosity and surface potential, making them efficient and low-resistance filters. The effects of adding CM on fiber diameter, pore size, porosity, roughness, and surface potential on filtration performance were investigated. The filtration performance of the materials, including the PM filtration capabilities, recirculation filtration, and loading test performance, was also studied. The preparation of self-curling melt-blown fibers represents a novel approach to creating high-efficiency and low-resistance air filters.

## 2. Materials and Methods

### 2.1. Materials

Polypropylene (PP) pellets (Y1500, 0.7386 g/cm^3^ density) with a melt flow rate (MFR) of 1800 g/10 min at 230 °C were purchased from China-Base Petrochemical Co., Ltd. (Ningbo, China) Crimped masterbatch (CNF01, 0.8288 g/cm^3^ density, MFR = 700 ± 100 g/10 min at 210 °C), referred to as CM, were supplied by Keimei Plastifizierung Technik (Yantai) Co., Ltd. (Yantai, China).

### 2.2. Fabrication of Melt Blown Nonwovens

Before the spinning, polypropylene and CM were dried in a vacuum drying oven at 80 °C for 8 h to remove moisture. The mixture of PP and CM in different proportions was evenly mixed for 30 min by a mixer. The weight ratio was PP:CM = 9:1, 8:2, 7:3. The samples prepared by pure PP and PP/CM mixture of different proportions are named CM–0, CM–1, CM–2, and CM–3, respectively.

**Figure 1:**
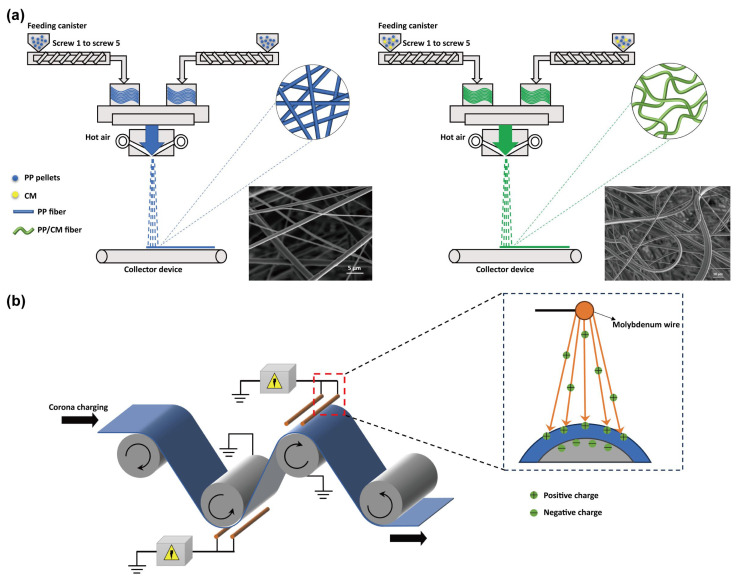
Fabrication of the melt-blown nonwovens. (**a**) Fabrication of PP/CM fibers. (**b**) Corona charging process.

The crimped melt-blown fibers were prepared with a twin-screw melt-blower (SMS300-Bico-M, KMD Plastifizierung Technik GmbH, Lübeck, Germany). Melt-blowing process parameters are listed in Table 1. The mixed polymer was placed into a hopper at a temperature of 80 °C and transported to the screw extrusion area. The polymer melted into a liquid state in the screw region and flowed to the spinning box by the shearing force. Eventually, the molten polymer converged at the die head and flowed out of the spinneret, where it was extruded by hot air to create a fiber. Fibers were spread onto the receiving device, and the basis weight of all samples was approximately 30 g/m^2^. The process of preparing melt-blown nonwovens is illustrated in Figure 1.

### 2.3. Corona Charging

The samples with CM–0, CM–1, CM–2, and CM–3 were treated with corona electret. Corona charging utilizes a high-voltage electric field to trigger air breakdown, resulting in a charge being deposited on the polymer surface or captured by its traps. This experiment utilized a double-sided corona charging device, which comprises a high-voltage power supply and two sets of electrode wires. The sample was positioned between the electrode wire and the substrate. After applying a high voltage, charges are deposited on both sides of the melt-blown material, resulting in a double-sided electret effect. The applied voltage and charging time were 60 kV and 10–30 s, respectively.

### 2.4. Characterization

The surface elements of feedstocks were analyzed by energy-dispersive X-ray spectrometer (EDS, Thermo Scientific Helios 5 CX, Thermo Fisher Scientific, Waltham, MA, USA). The Fourier transform infrared (FTIR) spectra of feedstocks were characterized via a Fourier transform infrared spectrometer (Thermo Fisher Nicolet Is5, Thermo Fisher Scientific, Waltham, MA, USA). The thermal stability information of the melt-blown nonwovens was obtained through a thermogravimetric analyzer (TA-Q500, TA Instruments, New Castle, DE, USA). The thermal behavior was studied via differential scanning calorimetry (TA-DSC25, TA Instruments, USA) in a nitrogen atmosphere. X-ray diffraction instrument (Rigaku Ultima IV, Rigaku Corporation, Tokyo, Japan) was used to characterize the crystallization of PP/CM blends. The test conditions were a wide-angle diffract-Cu target (5–90°) with a scanning rate of 2°/min. The melt flow index (MFR) of PP/CM blends was tested on a melt flow rate meter (BOS-300ZT, Boshi Testing Equipment Co., Ltd., Xiamen, China). 

The surface morphology of melt-blown nonwovens was investigated by scanning electron microscope (SEM) (TESCAN MIRA LMS, Tescan China Ltd., Shanghai, China) after sputter-coating the samples with gold. The fiber’s diameter and distribution were analyzed from more than 100 random fibers by the software Nano Measurer 1.2. The pore diameter of the melt-blown nonwovens was characterized by a capillary pore diameter analyzer (CFP-1500-AEXL, Porous Materials Inc., Ithaca, NY, USA). To observe the effect of fiber curl shape on the roughness of melt-blown fiber nonwovens, the surface roughness of melt-blown fiber nonwovens was measured by white light interference optical profilometer (Bruker Contour GT-K 3D, Bruker Corporation, Billerica, MA, USA). The surface potential of PP/CM nonwovens was tested on a non-contact electrostatic field meter (FMX-004, Simco Japan Inc., Kobe, Japan).

The thickness of melt-blown nonwovens was measured by a digital display thickness gauge (EVERTE CHYQFP25.4, Bon Agent Measuring Tools Co., Ltd., Shangqiu, China), and the average thickness was obtained by taking 20 different points on the sample. The porosity P of melt-blown nonwovens is calculated according to Equations (1) and (2).

(1) $$P=\left[1-\frac{s}{\rho \times t}\right]\times 100\%$$

(2) $$\rho =\rho_{pp}\times W_{pp}+\rho_{CM}\times W_{CM}$$

where *s* is the surface density of the sample, *t* is the sample thickness, *ρ* is the density of the sample, *ρ_pp_* is the density of PP, W*_pp_* is the proportion of PP mass, *ρ_CM_* is the density of CM, W*_CM_* is the proportion of CM mass.

### 2.5. Filtration Performances

The filtration performance and pressure drop of melt-blown nonwovens were measured by a particle filtration efficiency tester (DR251XL, Wenzhou Darong Textile Instrument Co., Ltd., Wenzhou, China) according to GB 2626-2019. The sodium chloride (NaCl) aerosol solution concentration was 2.0 wt%, and the particle diameter was normally distributed with a median diameter of 0.075 um and a geometric standard deviation of less than 1.86. Aerosol particles passed through the testing area of 100 cm^2^ at a flow rate of 85 L/min. The filtration efficiency was calculated by measuring the concentration of aerosol particles upstream and downstream of melt-blown nonwovens. The filtration efficiency (*η*) is calculated by the following equation:

(3) $$\eta =\frac{C_{up}-C_{down}}{C_{up}}\times 100\%$$

where C*_up_* and C*_down_* represent the concentration of aerosol particles in the upstream and downstream of melt-blown nonwovens, respectively.

The pressure drop is calculated by measuring the gas pressure difference between the upstream air inflow side and the downstream air outflow side of the filter material. The pressure drop (∆P) is calculated by the following formula:

∆P = P_1_ − P_2_ (4)

where P_1_ is the pressure before filtration and P_2_ is the pressure after filtration.

Since filtration efficiency and pressure drop are conflicting, neither of them is valid for assessing the filtration performance of melt-blown nonwovens alone, so the quality factor is used to comprehensively assess the filtration performance of the material. The larger the quality factor, the better the filtration performance of the material. The quality factor (*Q_f_*) is calculated by the following formula:

(5) $$Q_{f}=-\frac{\mathrm{Ln}\left(1-\;\eta \right)}{\Delta P}$$

The dust holding capacity and pressure drop growth rate are important indicators to characterize the service life of the filter material. A filtration loading test was performed to investigate the dynamic filtration property according to GB 2626-2019 and NIOSH Standards (Title 42 CFR Part 84) for N95. The dust holding capacity of melt-blown nonwovens was also evaluated by DR251XL, which is defined as the mass of particles deposited on the filter per unit area when the initial pressure drop doubles. The NaCl aerosol solution concentration was adjusted to 5.0 wt% and the air flow rate remained at 85 L/min. The dust holding capacity is calculated by the following equation:

(6) $$D=\frac{\left(m_{1}-m_{0}\right)}{A}$$

where *m*_1_ is the mass of the filter material after dust tolerance, *m*_0_ is the mass of the filter material, and *A* is the effective filter area of the filter material.

## 3. Results and Discussion

### 3.1. Chemical Composition Analysis

**Figure 2:**
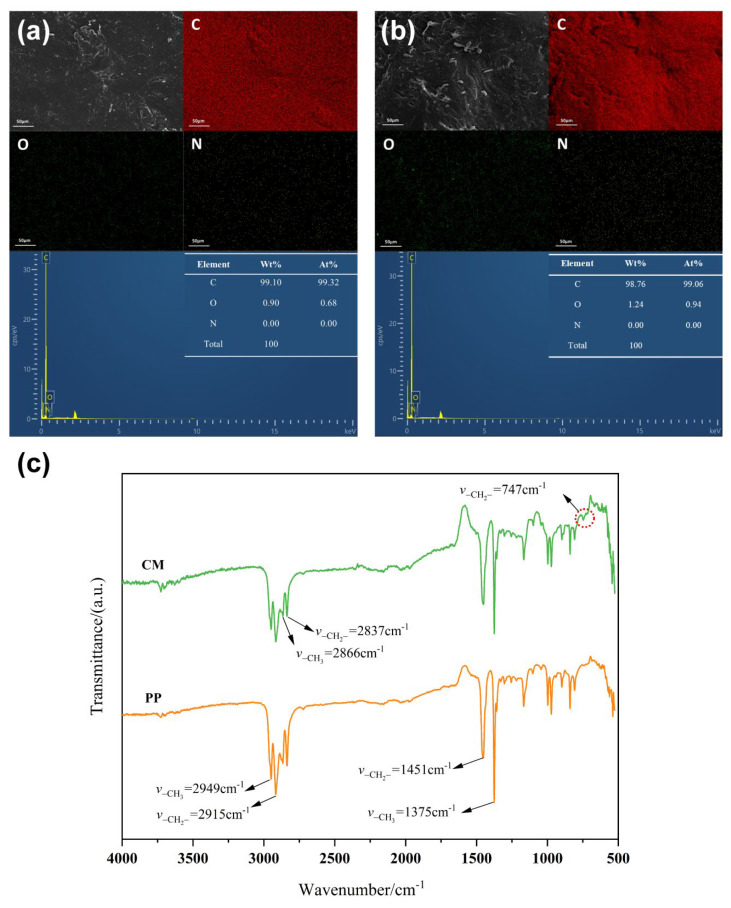
EDX images of (**a**) PP and (**b**) CM plastic, FT-IR spectra of (**c**) PP and CM plastic.

The content and distribution of C, O, and N elements in PP and CM were explored by an energy spectrum instrument, as shown in Figure 2a,b. EDX spectra showed that the carbon element was uniformly distributed on the entire surface of PP and CM, and the content was similar between these two species. This was consistent with the information provided by the supplier that CM was a class of curly masterbatch blended with PP and PE. To further validate this result, the chemical structure of PP and CM was investigated by FTIR in Figure 2c. From the results, the signal peaks at 2948 cm^−1^ and 2865 cm^−1^ were the stretching vibration carbon-hydrogen bond in –CH_3_. The signals at 2916 cm^−1^ and 2836 cm^−1^ were the stretching vibrations carbon-hydrogen bond in –CH_2_–. The bending vibration signals of –CH_2_– and –CH_3_ were 1457 cm^−1^ and 1375 cm^−1^, respectively [22,23,24]. The infrared spectra of PP and CM were less different, indicating that the main component in CM was still polypropylene. However, there was an oscillating vibration peak at 747 cm^−1^ from continuous methylene (*n* > 4) [25], which was not appeared in PP, indicating the presence of polyethylene in CM. 

### 3.2. Thermal Properties

**Figure 3:**
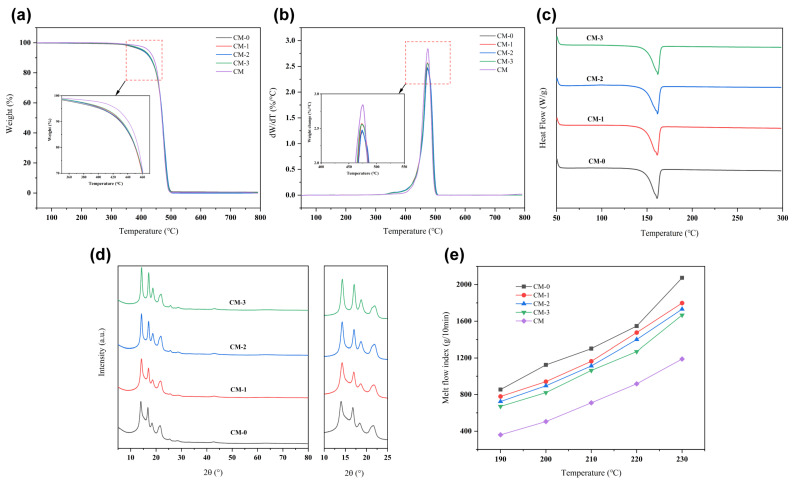
CM–0, CM–1, CM–2 and CM–3 (**a**) TG. (**b**) DTG. (**c**) DSC heating scans. (**d**) XRD patterns. (**e**) MFR at different temperatures.

Thermogravimetric analysis was conducted on CM plastics and PP/CM melt-blown fibers to evaluate their thermal stability. The results are presented in Figure 3a,b. The thermogravimetric (TG) curves indicate minimal degradation of CM plastic chips and PP/CM fibers up to 300 °C. However, there is a notable weight loss in the range of 350 and 500 °C due to the decomposition of the polymer. Based on the DTG curves presented in Figure 3b, it’s observed that all CM–0, CM–1, CM–2, CM–3, and CM exhibited the highest rates of decomposition at approximately 475 °C. At this temperature, the weight loss rates were 59.78%, 57.78%, 56.42%, 60.87%, and 65.37%, respectively. This stage is the main weightless phase of the polymer, where the macromolecular chains are heavily decomposed. As the amount of CM increased, the weight loss curve and decomposition temperature of PP changed less. This suggests that CM and PP share similar thermodynamic properties. The addition of CM has a minimal effect on the thermal performance of PP. The thermal degradation process of the PP/CM blends was found to be similar at different levels of CM content. 

Figure 3c displays the DSC melting curve of the PP/CM melt-blown fibers, illustrating the thermal behavior of PP at varying CM ratios. From the results, there is no significant difference in thermal performance between the PP/CM blends and pure PP. Compared to PP’s melting point of 161.04 °C, the introduction of CM results in no new melting peaks. This may be due to both PP and CM being non-polar crystalline polymers, and their similar structures result in good compatibility with each other [26]. During the crystallization process, the PP/CM polymer forms a mixed crystal, which is indicated by the appearance of a melting peak corresponding to the PP/CM polymer in the melting curve [26,27]. It is worth noting that the content of CM in PP/CM polymer is lower, and the melting peak does not exhibit significant deviation.

As depicted in Figure 3d, the XRD curve of CM–0 displays characteristic diffraction peaks at 2θ = 14.0°, 16.8°, 18.4° and 21.6°, which correspond to the (110), (040), (130), and (131) crystal faces of α-PP. These faces are part of the common α monoclinic crystal system [28,29,30]. After the introduction of crimped material, all diffraction peaks shift to the right, and CM–3 exhibits the largest degree of deviation. The results show that the lattice spacing of PP/CM is reduced, which further confirms the presence of PE in PP/CM fibers.

The melt flow rate (MFR) of PP/CM polymer is shown in Figure 3e. The MFR of different polymers increased as the temperature increased from 190 °C to 230 °C. The melt fluidity was positively correlated with the temperature. This phenomenon can be attributed to the fact that as the temperature rises, the intermolecular forces between the polymer molecules decrease, which in turn, increases the molecular chain motion. Thus, this leads to an increase in the melt flow rate. The MFR of PP increased from 855 g/10 min to 2074 g/10 min, while the MFR of CM only increased from 360 g/10 min to 1189 g/10 min. As the CM content increased, the melt flow rate of the PP/CM mixture decreased. This could be attributed to the higher molecular weight of the CM polymer and the increased degree of entanglement between polymer chains [31].

### 3.3. Microstructure Characterization

The melt-blown technology was utilized to prepare a filter that exhibited high filtration efficiency and ultra-low pressure drop. The aim was to investigate the impact of new curly melt-blown fibers on the filtration performance of particulate contaminants. The experiments were designed to optimize fiber structure based on the following criteria: (1) The fiber diameter and aperture should be small to enhance filtration efficiency. (2) The fiber should have a certain crimp and maintain high porosity to minimize filtration resistance. (3) The filter material should have a certain degree of looseness to increase dust capacity and improve service life. PP/CM melt-blown nonwovens with varying morphologies and structures were produced using low-viscosity polypropylene and high-viscosity coiled masterbatch.

**Figure 4:**
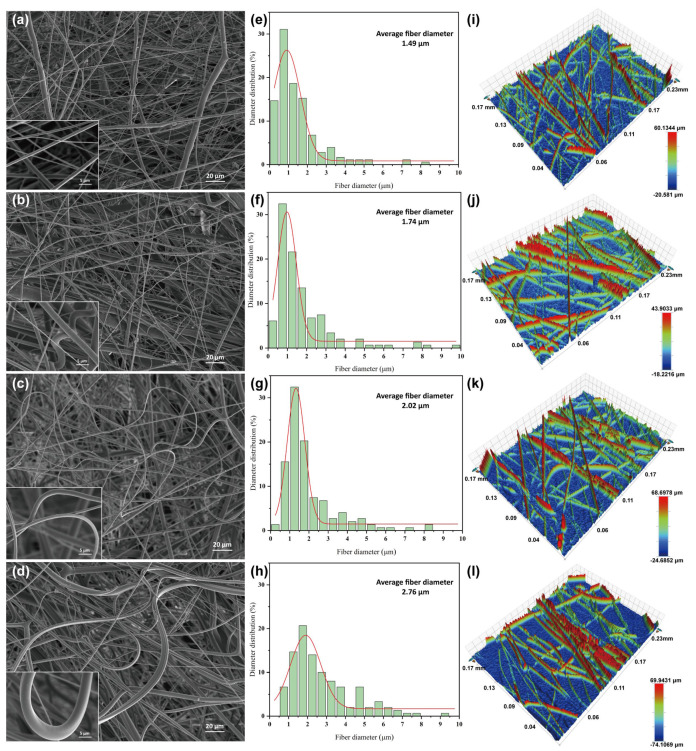
SEM images of PP/CM melt-blown webs of (**a**) CM–0, (**b**) CM–1, (**c**) CM–2, and (**d**) CM–3. Fiber diameter distribution of (**e**) CM–0, (**f**) CM–1, (**g**) CM–2, and (**h**) CM–3. Surface roughness profile of (**i**) CM–0, (**j**) CM–1, (**k**) CM–2, and (**l**) CM–3.

The melt-blown spinning performance of PP with varying levels of CM content was studied. The surface microscopic morphology was recorded by SEM, as presented in Figure 4a–d. CM–0 consists of melt-blown fibers made of pure polypropylene. In Figure 4a, it is evident that the fibers made of pure polypropylene are straight and cylindrical, and there are no visible kinks or curls in the bundles. Meanwhile, CM–1, CM–2, and CM–3 are melt-blown nonwovens prepared by incorporating 10%, 20%, and 30% of CM, respectively. The degree of fiber curl increases significantly with increasing CM content. When the amount of CM is minimal, the interaction force between PP and CM is insufficient to cause axial torsion in the fibers, resulting in the absence of a distinct curling structure. With an increase in the amount of CM, the longitudinal stress on the fiber by the two components also increases, resulting in the formation of a noticeable curling structure. The unique crimp structure can enhance the porosity and looseness of melt-blown nonwovens, effectively reducing filtration resistance. This highlights the significant role of crimp structure in filtering fibers.

Fiber crimping is primarily attributed to differences in compatibility and viscosity between PP and CM. Polypropylene (PP) and polyethylene (PE) are likely to be the primary components of materials used for fiber curling. Both polypropylene (PP) and polyethylene (PE) are straight-chain hydrocarbon polymers. These materials are non-polar and crystalline, and they have a similar chemical structure, which makes them compatible with each other. During the melt-blowing process, low-viscosity PP and high-viscosity crimped materials generate longitudinal stress in the fiber section. This stress causes the fiber to deviate from its axial torsion, potentially resulting in a three-dimensional crimp structure [32]. Although the crimped melt-blown fibers were produced, the degree and amount of crimping were still low. Initially, only a small amount of CM was blended with PP to preserve the fundamental properties of the melt-blown fibers. This resulted in a reduction of stress within the fiber cross-section caused by the PP/CM bicomponent. As a result, the fibers are curled in an S-shape and have fewer kinks. Secondly, due to the direct blending of the polymer masterbatch, a significant portion of the straight fibers consists of either PP or CM fibers rather than PP/CM bicomponent fibers. Therefore, introducing high-viscosity polymers into polypropylene or using pelletization to enhance the homogeneous blending of polymer bicomponent can be effective methods for increasing the curl structure of the fibers.

Figure 4e–h and Table 2 show the fiber diameters of melt-blown nonwovens with different CM contents. The results showed that the fiber diameters were normally distributed, with all being less than 10 µm [33]. As the CM content increased, there was a tendency for the fiber diameter to increase. The average diameters of PP, CM–1, CM–2, and CM–3 were 1.49, 1.74, 2.02, and 2.76 μm, respectively. Notably, CM–3 had the widest distribution of fiber diameters. This is determined by the melt flow rate of the polymer [34,35]. The MFR of PP is 1800 g/10 min, and the polymer has a low viscosity, which makes it easy to stretch and form ultrafine fibers. After blending with CM, the melt viscosity of the polymer increases while its flowability decreases. This change is not conducive to stretching and refining. In addition, while it is possible to adjust the air pressure and DCD to reduce the fiber diameter of CM–2 and CM–3, increasing the air pressure can cause “splashing” [36]. Similarly, increasing the DCD can reduce the bonding force between the fibers, ultimately weakening the strength of the melt-blown nonwovens [37]. The filtration performance of melt-blown nonwovens is affected by different fiber diameters and distributions. In general, thicker fibers tend to have lower filtration performance in nonwovens, but the curl of the fiber compensates for this.

Roughness measurements were made on a randomly selected x-axis or y-axis in a certain area of melt-blown nonwoven. The fiber surface roughness of melt-blown nonwovens was characterized by measuring the mean square roughness (Rq) of the samples. The test results are shown in Table 2 and Figure 4i–l. The results showed that the roughness of the fiber web increases with the CM content. The roughness of CM–3 was the highest at 14.37 μm, which is almost 50% greater than that of the pure PP fiber at 7.81 μm. PP has the flattest fiber surface, with a distance of 80.72 μm between the highest and lowest points. The addition of curl material, especially CM–3, increased the variation in fiber surface roughness. The distance between the highest and lowest points reached 144.05 μm, which was 1.78 times higher than that of the PP fiber. The fiber morphology profile shown in Figure 3 is consistent with the information presented in Table 2, providing additional evidence that a significant proportion of CM increases the curl of the fiber, resulting in increased roughness [15,38].

**Figure 5:**
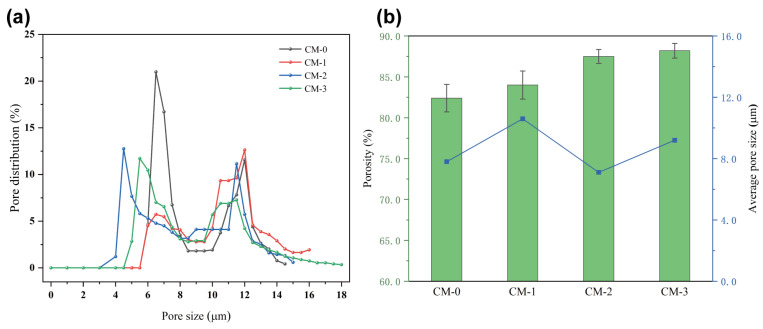
PP/CM melt-blown nonwovens (**a**) Pore size distribution and (**b**) Average pore size and porosity.

The pore size and size distribution of PP/CM melt-blown nonwovens were statistically analyzed, and the results are illustrated in Figure 5a. It is evident that the aperture of PP/CM melt-blown nonwovens changes when CM is introduced. The pore size distribution of the resulting filter exhibits a bimodal distribution, which is strongly influenced by the flow properties of the spinning melt and the precise control of the spinning process parameters. The pore size of CM–1 melt-blown nonwovens is mainly concentrated in the range of 11~13 μm, while the pore size of CM–0, CM–2, and CM–3 is less than 10 μm. Obviously, CM–1 has the largest average pore size (~10.6 μm), and CM–2 has the smallest average pore size (~7.1 μm). The incorporation of curly fibers not only improves the pore size structure and distribution but also increases the porosity of PP melt-blown nonwovens. 

Figure 5b displays the porosity variations among various melt-blown nonwoven fabrics. It is observed that the porosity of CM–1 melt-blown fiber material (84.0%) has no significant change compared with that of PP (82.4%). After increasing the proportion of CM, the porosity of CM–2 (87.5%) was significantly higher than that of PP and CM–1 [39]. This can be attributed to the increased degree of fiber curl. When the CM content was at 30%, the porosity of CM–3 only showed a negligible increase of 0.7%. This could be attributed to the thickening fiber diameter. The intrinsic mechanism for enhancing porosity is due to the curled fiber morphology, which expands the occupied volume of a single fiber from a two-dimensional scale to a three-dimensional scale, resulting in a spatial support effect. In general, melt-blown fiber filter materials that have high porosity and multi-scale pore sizes are necessary to achieve both high efficiency and low resistance.

### 3.4. Surface Potential 

**Figure 6:**
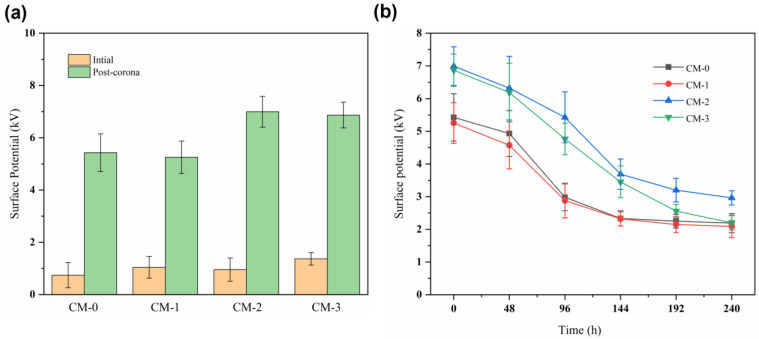
The surface potential of melt-blown nonwovens after (**a**) initial and corona and (**b**) indoor storage for 240 h.

To investigate the impact of the curling masterbatch on the electrostatic characteristics of PP/CM melt-blown nonwovens, the surface potential of the samples was measured. As depicted in Figure 6, the surface potentials of CM–0, CM–1, CM–2, and CM–3 are 5.43, 5.25, 6.99, and 6.87 kV, respectively, under identical conditions. The inclusion of CM elevated the surface potential of the PP nonwovens significantly. During the electret process, electric charges are deposited onto the fiber surface through an electric field and then captured by the material’s traps [40]. The “curling” effect of CM on PP fibers increases the thickness and porosity of the nonwovens. This, in turn, helps to trap and retain charged particles, thereby increasing the surface potential [41].

During the process of charge decay, the surface charge of the nonwovens decreased rapidly in the first 144 h and then gradually slowed down and stabilized thereafter. The attenuation of surface potential is significantly higher for CM–2 (6.99 kV to 2.96 kV) and CM–3 (6.87 kV to 2.20 kV) compared to CM–0 (5.43 kV to 2.19 kV). This is because the melt-blown webs absorb a large amount of charge on the fiber surface after electret. The moisture in the air neutralizes the charge on the surface, leading to a rapid decay of the surface charge until it reaches a stable state [42]. CM–2 and CM–3 are fibrous materials with convoluted structures and large open pores. These features increase the likelihood of contact between the charge and water molecules in the air, thereby reducing their surface potential. Ultimately, CM–2 exhibited the highest surface electrostatic potential, likely attributable to its greater porosity, smaller fiber diameter, and pore size.

### 3.5. Filtration Efficiency

**Figure 7:**
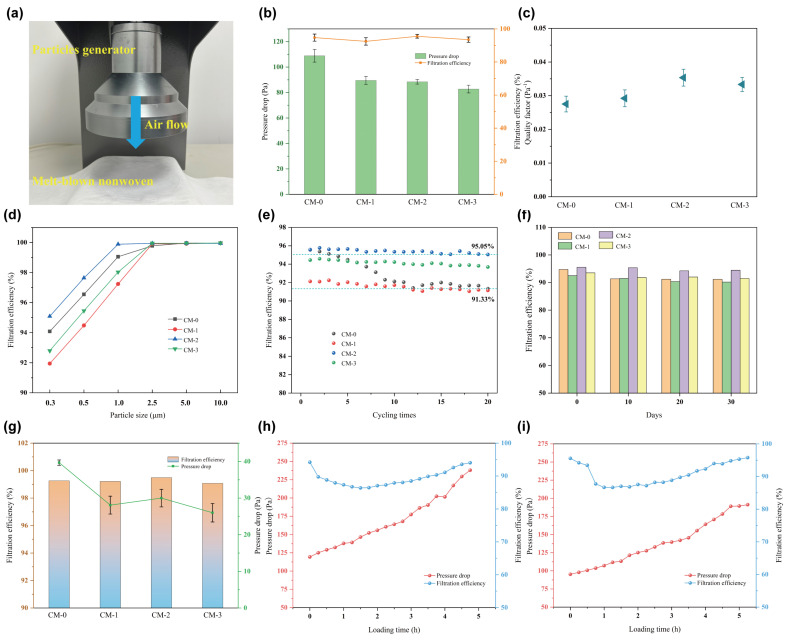
(**a**) Diagram of air filtration equipment used to test the filtration efficiency. (**b**) The filtration efficiency and pressure drop of melt-blown nonwovens for PM_0.3_ under 85 L min^−1^. (**c**) Quality factor of melt-blown nonwovens. (**d**) Filtration efficiency versus particle size. (**e**) Cycling filtration performance of melt-blown nonwovens for PM_0.3_ under 85 L min^−1^. (**f**) Decay of filtration efficiency within 30 days. (**g**) Filtration efficiency and pressure drop of melt-blown nonwovens for PM_0.3_ under 32 L min^−1^. (**h**) Filtration efficiency and pressure drop of CM–0 during loading. (**i**) Filtration efficiency and pressure drop of CM–2 during loading. The detection particle size is ~0.3 μm in subfigures (**h**) and (**i**).

Melt-blown nonwovens exhibit high filtration efficiency, which enables them to effectively capture airborne particulate matter (PM). However, they also have high-pressure drop values, which means they can cause significant respiratory resistance. The balance between filtration efficiency and pressure drop is crucial when studying filtration materials. As shown in Figure 7a, a particle filtration efficiency tester was used to evaluate the effectiveness of melt-blown nonwovens in removing PM. The filtration efficiency is calculated by measuring the concentration of PM (particulate matter) particles upstream of the generator and downstream of the receiving sensor. The filtration efficiency of PP/CM melt-blown nonwovens against PM_0.3_ was extensively investigated at a flow rate of 85 L/min. Filtration efficiency, pressure drop, and quality factor (QF) were used as evaluation parameters [43]. The filtration efficiencies of CM–0, CM–1, CM–2, and CM–3 were 94.74%, 92.47%, 95.54%, and 93.51%, respectively. It can be found that compared to PP, the filtration efficiency of both CM–1 and CM–3 reduced to about 93%, while the filtration efficiency of CM–2 fiber is improved while obtaining a lower pressure drop, which is conducive to high-performance air filtration. In general, both reducing the fiber diameter and increasing the surface charge of nonwovens can effectively improve filtration efficiency. However, the unique curl morphology and fluffy aggregate structure of PP/CM fibers have a major impact on particle retention. During filtration, the fiber diameter and crimped structure work together to affect filtration efficiency and pressure drop. The dominant factor for CM–1 is the fiber diameter. The larger fiber diameter of CM–1 (1.74 μm) compared to CM–0 (1.49 μm) reduces the specific surface area, thereby decreasing the probability of PM capture and leading to a reduction in filtration efficiency. In the case of CM–3, the increase in fiber diameter and fluffiness of the fiber aggregates results in a smoother passage of PM with the airflow, reducing the likelihood of PM being captured and leading to a decrease in filtration efficiency. The crimped structure of the fibers increases the filter thickness and prolongs the filtration time of the particles through the material. This, in turn, enhances the likelihood of the particles being trapped by the fibers [44,45]. From the results, CM–2 achieved excellent filtration efficiency by balancing the combined effect of fiber diameter and crimped structure. 

During the filtration process, the pressure drop generated by the airflow through the melt-blown nonwoven is solely influenced by its pore structure. The corona electret treatment has less effect on the structure of nonwovens. As depicted in the histogram in Figure 7b, the pressure drops decrease by approximately a quarter, from 108.88 to 82.74 Pa, with an increase in the percentage of CM. CM–0 fibers have the smallest average diameter (1.49 μm), which significantly increases the specific surface area of the fibers. However, this also reduces the air slip effect and increases the resistance. Generally speaking, increasing the thickness of fiber mesh results in an increase in filtration pressure drop. However, increasing the thickness of CM–2 and CM–3 is not achieved by increasing the density of the fiber mesh. Instead, it is the spatial support effect of the crimped fiber that creates a loose and porous structure, providing the special advantage of low-pressure drop [4]. Furthermore, the quality factor (QF) measures the collective filtration efficacy of melt-blown nonwovens by assessing the correlation between filtration efficiency and pressure drop. Figure 7c displays the comparative QF results of PP/CM melt-blown nonwoven for filtering PM_0.3_. Compared to other samples, CM–2 has a high QF (0.035 Pa^−1^) and shows potential for use in air filtration.

To evaluate the efficacy of PP/CM melt-blown nonwovens under real atmospheric conditions, the filtration performance for PM_0.3_, PM_0.5_, PM_1.0_, PM_2.5_, PM_5.0_ and PM_10.0_ were conducted. As shown in Figure 7d, the filtration efficiency of all melt-blown nonwovens significantly increased with the particle size. CM–2 exhibited superior performance compared to other samples when the PM was less than 2.5 μm. Furthermore, all samples achieved a filtration efficiency of 99.9% for PM greater than 2.5 μm. The recycling performance of PP/CM melt-blown nonwovens was also evaluated, and the results are presented in Figure 7e. After 20 filtration cycles, the filtration efficiency of CM–0 gradually decreased from 95.56% to 91.33%. In contrast, CM–2 maintained a consistently high filtration efficiency (95.58% to 95.05%). These results indicate that adding CM to PP melt-blown nonwoven enhances its filtration performance and practicality. Post-electret melt-blown nonwovens exhibit strong electrostatic adsorption of PM during the initial stage. However, the duration of high-performance PM adsorption is short-lived, and prolonged use can lead to a decrease in PM removal efficiency. Fortunately, the CM–2 not only exhibited excellent cycling performance but also maintained good stability during the 30-day filtration efficiency test. The filtration efficiency for PM_0.3_ remained consistent at 94% even after 30 days (Figure 7f). The filtration efficiency of the nonwovens for sodium chloride aerosol (PM_0.3_) was tested at a flow rate of 32 L/min, as shown in Figure 7g. The filtration efficiency of the nonwovens can reach 99% at low flow rates. At this point, the CM–2 still maintains an extremely low filter resistance of 30 Pa.

Figure 7h–i shows the load experiments of CM–0 and CM–2, respectively, and compares their dust-holding capacity. The dust capacity of CM–0 and CM–2 is 9.20 g/m^2^ and 10.39 g/m^2^, respectively. The basis weight of both is approximately 30 g/m^2^, while the dust capacity of CM–2 reaches a relatively high level. This is because CM–0 primarily intercepts particles on the surface to form a dense filter cake structure, while CM–2 can rely on its porous structure to capture a significant number of particles internally [46]. In addition, CM–2 takes longer to reach twice the initial resistance, indicating that CM–2 has a superior service life compared to CM–0.

### 3.6. Filtration Mechanism

**Figure 8:**
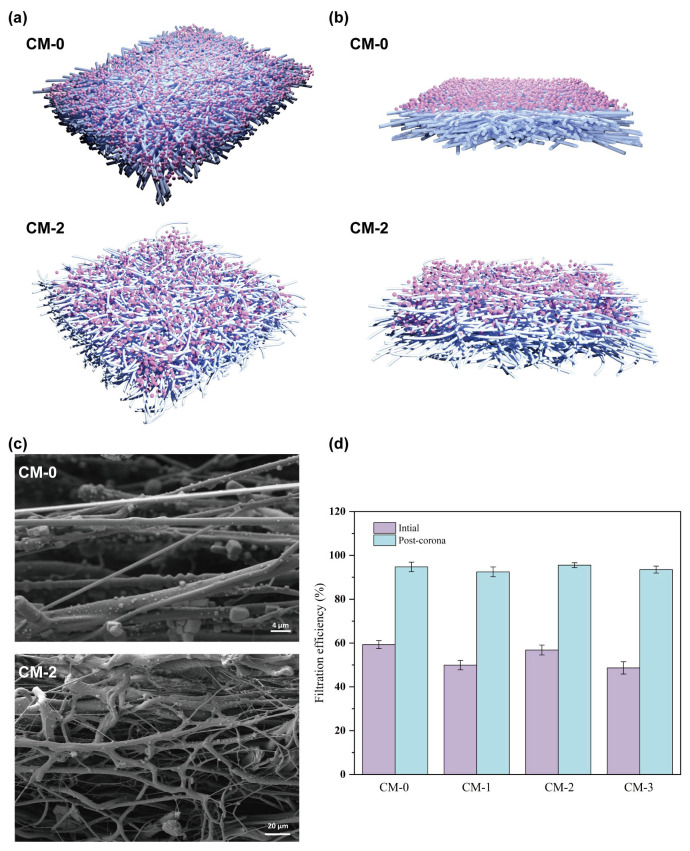
Particle loading on CM–0 and CM–2 fibers in (**a**) surface view, (**b**) cross-section view, and (**c**) SEM. (**d**) Filtration efficiency of melt-blown nonwovens for PM_0.3_ under 85 L min^−1^ after initial and corona. (Subfigures (**a**,**b**) were drawn using 3ds Max 2018 software).

The initial filtration phase of melt-blown nonwovens is called the steady-state phase when the five effects of particle capture by the mesh include physical interception, Brownian diffusion, inertial deposition, gravitational sedimentation, and electrostatic adsorption [47,48,49,50]. Among them, the electrostatic effect is the “active adsorption” way to achieve particle trapping, and the other four effects are the “passive trapping” of particles by a single fiber. As shown in Figure 8d, the filtration efficiency of CM–0 before the electret was 59.28%, which was slightly higher than that of CM–2 (56.81%). Combined with the surface potential analysis in 3.4, the melt-blown nonwovens showed higher and more stable filtration efficiency of CM–2 after corona charging. It can be seen that the crimped fibers enhanced the filtration mechanism of electrostatic adsorption. The incorporation of CM resulted in changes in the spinning performance, fiber diameter, and fiber web structure of polypropylene. This is because when CM is embedded in the polypropylene polymer, the fibers curl after the melt-spun fibers cool down. This curling is caused by localized, uneven shrinkage that occurs due to variations in thermal contraction between the polymers. The curled fibers create a fluffy structure in the fiber network and modify the distribution of pore sizes within it. As shown in Figure 8a–c, the conventional PP melt-blown fibers are cylindrical and have a smooth surface, and the fibers are only laid in a two-dimensional direction. The fibers are arranged in a random orientation in the plane. Under high-speed airflow, PM is physically intercepted by the surface of the fiber network, and the resistance increases rapidly. CM–2 melt-blown fibers have a convoluted structure, which is isotropic not only in the two-dimensional direction but also provides a richly curved three-dimensional spatial channel in the thickness direction. On the one hand, the thickness prolongs the filtration time of particles in the material and increases the probability of contact between fibers and particles, thus preventing PM from passing through the fiber network. On the other hand, the rich and tortuous three-dimensional space channels can make the air flow more smoothly through the fiber assembly, thus reducing filtration resistance. Therefore, PP/CM melt-blown fibers with the convoluted structure are expected to replace traditional rod-shaped fibers for efficient and breathable air filtration.

## 4. Conclusions

In summary, self-curling melt-blown nonwovens with high filtration efficiency and ultralow pressure drop were successfully fabricated based on PP and CM. When CM is embedded in the polypropylene polymer, the fibers curl after the melt-spun fibers cool down. This curling was caused by the uneven shrinkage that occurs due to variations in thermal contraction between the polymers. The curled fibers create a fluffy structure in the fiber network and modify the distribution of pore sizes within it. The difference between these two polymers creates a “torsion effect” on the fiber cross-section, resulting in the formation of three-dimensional crimped fibers. An increasing CM content leads to an extension of the fiber’s curl and fluffiness but with a coarsening of the fiber size. Under the same filtration conditions of sodium chloride aerogel, CM–2 exhibited similar filtration efficiency (95.54% vs. 94.74%), lower filtration resistance (88.68 Pa vs. 108.88 Pa), higher quality factor (0.035 Pa^−1^ vs. 0.028 Pa^−1^) and better dust holding capacity (10.39 g/m^2^ vs. 9.20 g/m^2^) compared to CM–0. After 30 days of indoor storage, the filtration efficiency of CM–2 remains above 94%. It is generally more desirable for consumers and mask manufacturers to maintain high filtration efficiency and minimize pressure drop. It’s believed that the self-curling melt-blown fibers developed in this work would be applied in the field of comfortable protective masks.

## Figures and Tables

**Table 1:** Processing conditions of melt-blown nonwovens.

Screw/°C	Die/°C	Air/°C	Pump Flow cc/min	Air Fan Power/Hz	DCD */mm
260	CM–0, CM–1: 270 CM–2, CM–3: 280	280	50	CM–0, CM–1: 20 CM–2, CM–3: 22	CM–0, CM–1: 260 CM–2: 220 CM–3: 180

* DCD is the distance from the spinning die to the fiber collector.

**Table 2:** Roughness profile of melt-blown nonwovens.

Roughness	CM–0	CM–1	CM–2	CM–3
Ra (μm)	5.34	4.54	7.62	9.93
Rq (μm)	7.81	6.47	10.77	14.37

## Data Availability

The data presented in this study are available on request from the corresponding author.
